# Tris(2-meth­oxy­ethanaminium) dodeca­molybdophosphate trihydrate

**DOI:** 10.1107/S1600536810028801

**Published:** 2010-07-24

**Authors:** Akbar Raissi Shabari, Sara Kharaghani, Mehrdad Pourayoubi

**Affiliations:** a Faculty of Chemistry, Islamic Azad University-North Tehran Branch, Tehran, Iran; b Department of Chemistry, Ferdowsi University of Mashhad, Mashhad, 91779, Iran

## Abstract

The asymmetric unit of the polyoxidometalate-based organic–inorganic hybrid title compound, (C_3_H_10_NO)_3_[PMo_12_O_40_]·3H_2_O, consists of one α-Keggin-type [PMo_12_O_40_]^3−^ polyoxido­anion, three independent [CH_3_–O–CH_2_–CH_2_–NH_3_]^+^ cations and three solvent water mol­ecules. The polyoxidoanion shows characteristic features with respect to bond lengths and angles. In the crystal structure, extensive inter­molecular N—H⋯O and O—H⋯O hydrogen bonding between the organic cations, inorganic anions and solvent water mol­ecules leads to a three-dimensional supra­molecular network.

## Related literature

For background information on polyoxometalate-based organic–inorganic hybrid materials, see: Pourayoubi & Mahjoub (2007 ▶, 2010 ▶); Raissi Shabari *et al.* (2009 ▶). For related structures, see: Gong *et al.* (2006 ▶); Han *et al.* (2005 ▶).
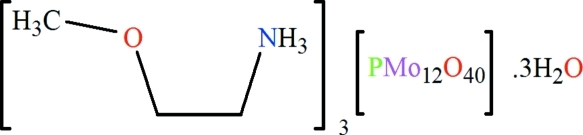


## Experimental

### 

#### Crystal data

(C_3_H_10_NO)_3_[PMo_12_O_40_]·3H_2_O
*M* *_r_* = 2104.66
Monoclinic, 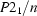

*a* = 12.7806 (2) Å
*b* = 27.0489 (4) Å
*c* = 14.6360 (2) Å
β = 114.876 (1)°
*V* = 4590.24 (12) Å^3^
*Z* = 4
Mo *K*α radiation
μ = 3.32 mm^−1^
*T* = 100 K
0.10 × 0.05 × 0.03 mm

#### Data collection

Bruker SMART APEXII CCD area-detector diffractometer
Absorption correction: multi-scan (*SADABS*; Bruker, 2005 ▶) *T*_min_ = 0.820, *T*_max_ = 0.907
100492 measured reflections
12174 independent reflections
10404 reflections with *I* > 2σ(*I*)
*R*_int_ = 0.042

#### Refinement

*R*[*F* ^2^ > 2σ(*F* ^2^)] = 0.022
*wR*(*F* ^2^) = 0.052
*S* = 1.00
12174 reflections
646 parameters
H-atom parameters constrained
Δρ_max_ = 0.66 e Å^−3^
Δρ_min_ = −0.69 e Å^−3^

### 

Data collection: *APEX2* (Bruker, 2005 ▶); cell refinement: *SAINT* (Bruker, 2005 ▶); data reduction: *SAINT*; program(s) used to solve structure: *SHELXTL* (Sheldrick, 2008 ▶); program(s) used to refine structure: *SHELXTL*; molecular graphics: *SHELXTL*; software used to prepare material for publication: *SHELXTL*.

## Supplementary Material

Crystal structure: contains datablocks I, global. DOI: 10.1107/S1600536810028801/lh5078sup1.cif

Structure factors: contains datablocks I. DOI: 10.1107/S1600536810028801/lh5078Isup2.hkl

Additional supplementary materials:  crystallographic information; 3D view; checkCIF report

## Figures and Tables

**Table 1:** Hydrogen-bond geometry (Å, °)

*D*—H⋯*A*	*D*—H	H⋯*A*	*D*⋯*A*	*D*—H⋯*A*
O1*W*—H1*WA*⋯O2*W*	0.75	2.00	2.726 (5)	161
O1*W*—H1*WB*⋯O31^i^	0.85	2.14	2.936 (4)	157
O2*W*—H2*WB*⋯O15	0.83	1.99	2.807 (3)	167
O2*W*—H2*WA*⋯O22^ii^	0.90	2.03	2.780 (3)	140
O3*W*—H3*WA*⋯O40^iii^	0.88	2.05	2.904 (3)	162
O3*W*—H3*WB*⋯O10^iv^	0.84	1.97	2.772 (3)	159
N1*S*—H1*NA*⋯O3*W*	0.91	1.93	2.812 (4)	164
N1*S*—H1*NB*⋯O2*S*^v^	0.91	2.59	3.182 (4)	123
N1*S*—H1*NB*⋯O9^v^	0.91	2.24	2.949 (4)	134
N1*S*—H1*NC*⋯O1*S*	0.91	2.31	2.731 (4)	108
N1*S*—H1*NC*⋯O31^v^	0.91	2.48	3.110 (4)	127
N1*S*—H1*NC*⋯O35^ii^	0.91	2.28	2.887 (3)	124
N2*S*—H2*NA*⋯O2*S*	0.91	2.42	2.814 (5)	106
N2*S*—H2*NA*⋯O8	0.91	1.94	2.811 (3)	159
N2*S*—H2*NB*⋯O1*W*	0.91	1.83	2.629 (5)	145
N2*S*—H2*NC*⋯O3*S*	0.91	1.93	2.798 (5)	158
N3*S*—H3*NA*⋯O23^ii^	0.91	2.31	3.110 (4)	146
N3*S*—H3*NB*⋯O34^iv^	0.91	2.08	2.955 (4)	161
N3*S*—H3*NC*⋯O2*W*	0.91	1.96	2.822 (4)	158

Symmetry codes: (i) 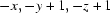; (ii) 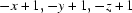; (iii) 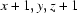; (iv) 
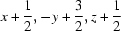
; (v) 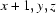.

**Fig. 1.**
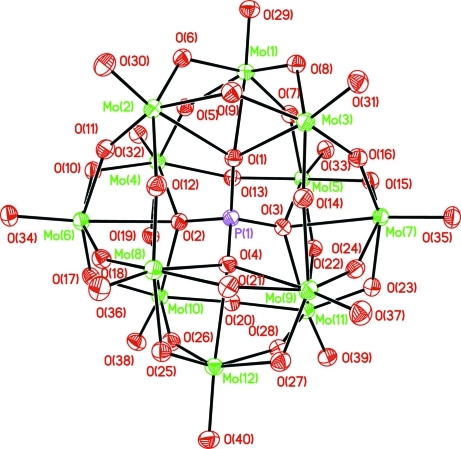
An ORTEP-style plot of the α-Keggin-type PMo12O403- polyoxoanion. Ellipsoids are given at the 50% probability level.

**Fig. 2.**
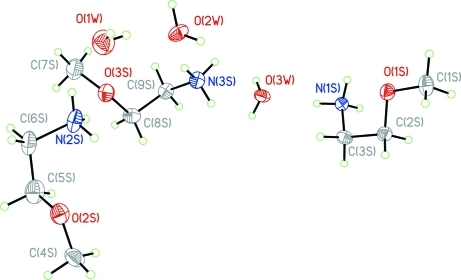
A view of the three symmetrically independent organic cations and the solvent water molecules. Ellipsoids are given at the 50% probability level.

## supplementary crystallographic information

### Comment

Keggin-type polyoxoanions have been used as the inorganic building blocks to construct polyoxometalate-based organic-inorganic hybrid materials containing the organic cations such as protonated-amino acid (Raissi Shabari *et al.*, 2009), heterocyclic base (Pourayoubi & Mahjoub, 2007) and amide (Pourayoubi & Mahjoub, 2010).

We report here on the synthesis and crystal structure of a new POM-based hybrid material containing a protonated aminoether.

The title polyoxometalate-based organic-inorganic hybrid compound consists of one [PMo_12_O_40_]^3-^ polyoxoanion, three symmetrically independent CH_3_–O–CH_2_–CH_2_–NH_3_^+^ cations and three solvent water molecules. The inorganic anion shows a classical α-Keggin structure (Fig. 1) with 4 different types of O atoms. This includes 12 terminal O atoms, 4 O atoms which are bonded to P and Mo, 12 MoO_6_ octahedra corner-shared and 12 MoO_6_ octahedra edge-shared oxygen atoms.

The central PO_4_ tetrahedron is slightly distorted and is surrounded by 12 distorted MoO_6_ octahedra. The P—O bond lengths range from 1.534 (2) to 1.539 (2) Å, and the O—P—O angles are in the range of 109.20 (11)–109.66 (11)°.

All three organic cations (Fig. 2) show slight differences in bond lengths, angles and torsion angles. They are involved in an extensive hydrogen bonding. Several N–H···O (N···O distances are in the range of 2.629 (5) to 3.182 (4) Å) and O–H···O (O···O distances are in the range from 2.726 (5) to 2.936 (4) Å) hydrogen bonds between the organic cations, inorganic anions and crystal water molecules lead to a 3-D supramolecular network.

In the title hybrid compound, the position of organic cations and water molecules allows to direct interaction of one polyoxoanion with four neighboring polyoxoanions via O···O contacts [2.80-3.018 Å]. Two important O···O contacts are the O36···O6 (2.800 Å) and O32···O17 (2.814 Å) interactions, O36 and O32 are the terminal and O6 and O17 are the bridged oxygen atoms of the polyoxoanions. The less important O···O contacts are O31···O35 (3.018 Å), O29···O34 (3.012 Å), O27···O28 (2.981 Å) and O24···O40 (2.915 Å) [O31, O35, O29, O34 and O40 are terminal and O24, O27 and O28 are bridging oxygen atoms]. Similar interactions have been observed in the structure of (C_5_N_2_H_7_)_5_H[P_2_Mo_5_O_23_] (C_5_N_2_H_7_ = protonated 2-aminopyridine) reported by Gong *et al.* (2006) [O_terminal_···O_terminal_ = 2.398 Å] and in the structure of (H_3/4_pbpy)_4_[PMo_12_O_40_].1.25H_2_O (pbpy = 5-phenyl-2-(4-pyridinyl)pyridine) reported by Han *et al.* (2005) [O_terminal_···O_terminal_ and O_terminal_···O_bridge_ in the range of 2.931 to 3.001 Å].

### Experimental

The title hybrid compound was obtained from mixing H_3_PMo_12_O_40_ and SrCl_2_.2H_2_O and then the treatment with CH_3_OCH_2_CH_2_NH_2_ in a mixture of H_2_O and CH_3_CN. ^31^P-NMR (DMSO-d_6_, p.p.m.): -4.11. IR (KBr, cm^-1^): 3371.4 m, 2908.7 w, 1611.7 m, 1501.6 m, 1208.2 w, 1062.1 s, 957.9 vs, 878.8 s, 783.6 vs.

### Refinement

The hydrogen atoms of NH_3_ groups were included in calculated positions with N-H = 0.91Å and those of the H_2_O molecules were found in a difference Fourier synthesis. The H(C) atom positions were placed in calculated positions with C-H = 0.98-0.99Å. All hydrogen atoms were refined in isotropic approximation in a riding-model with the *U*_iso_(H) parameters in the range 1.2-1.5 *U*_eq_(C,N,O).

### Crystal data

**Table:**

(C_3_H_10_NO)_3_[PMo_12_O_40_]·3H_2_O	*F*(000) = 3992
*M**_r_* = 2104.66	*D*_x_ = 3.045 Mg m^−^^3^
Monoclinic, *P*2_1_/*n*	Mo *K*α radiation, λ = 0.71073 Å
Hall symbol: -P 2yn	Cell parameters from 3521 reflections
*a* = 12.7806 (2) Å	θ = 2–25°
*b* = 27.0489 (4) Å	µ = 3.32 mm^−^^1^
*c* = 14.6360 (2) Å	*T* = 100 K
β = 114.876 (1)°	Prism, light-green
*V* = 4590.24 (12) Å^3^	0.10 × 0.05 × 0.03 mm
*Z* = 4	

### Data collection

**Table:**

Bruker SMART APEXII CCD area-detector diffractometer	12174 independent reflections
Radiation source: fine-focus sealed tube	10404 reflections with *I* > 2/s(*I*)
graphite	*R*_int_ = 0.042
φ and ω scans	θ_max_ = 29.0°, θ_min_ = 1.7°
Absorption correction: multi-scan (*SADABS*; Bruker, 2005)	*h* = −17→17
*T*_min_ = 0.820, *T*_max_ = 0.907	*k* = −36→36
100492 measured reflections	*l* = −19→19

### Refinement

**Table:**

Refinement on *F*^2^	Primary atom site location: structure-invariant direct methods
Least-squares matrix: full	Secondary atom site location: difference Fourier map
*R*[*F*^2^ > 2σ(*F*^2^)] = 0.022	Hydrogen site location: mixed
*wR*(*F*^2^) = 0.052	H-atom parameters constrained
*S* = 1.00	*w* = 1/[σ^2^(*F*_o_^2^) + (0.026*P*)^2^ + 6.*P*] where *P* = (*F*_o_^2^ + 2*F*_c_^2^)/3
12174 reflections	(Δ/σ)_max_ = 0.003
646 parameters	Δρ_max_ = 0.66 e Å^−^^3^
0 restraints	Δρ_min_ = −0.69 e Å^−^^3^

### Special details

**Table:**

Geometry. All e.s.d.'s (except the e.s.d. in the dihedral angle between two l.s. planes) are estimated using the full covariance matrix. The cell e.s.d.'s are taken into account individually in the estimation of e.s.d.'s in distances, angles and torsion angles; correlations between e.s.d.'s in cell parameters are only used when they are defined by crystal symmetry. An approximate (isotropic) treatment of cell e.s.d.'s is used for estimating e.s.d.'s involving l.s. planes.
Refinement. Refinement of *F*^2^ against ALL reflections. The weighted *R*-factor *wR* and goodness of fit *S* are based on *F*^2^, conventional *R*-factors *R* are based on *F*, with *F* set to zero for negative *F*^2^. The threshold expression of *F*^2^ > σ(*F*^2^) is used only for calculating *R*-factors(gt) *etc*. and is not relevant to the choice of reflections for refinement. *R*-factors based on *F*^2^ are statistically about twice as large as those based on *F*, and *R*- factors based on ALL data will be even larger.

### Fractional atomic coordinates and isotropic or equivalent isotropic displacement parameters (Å^2^)

**Table:**

	*x*	*y*	*z*	*U*_iso_*/*U*_eq_	
Mo1	0.24736 (2)	0.664083 (9)	0.512361 (18)	0.01740 (5)	
Mo2	−0.00825 (2)	0.715144 (9)	0.349457 (19)	0.01756 (5)	
Mo3	0.00282 (2)	0.592678 (9)	0.412826 (19)	0.01770 (5)	
Mo4	0.35509 (2)	0.720875 (9)	0.337823 (18)	0.01669 (5)	
Mo5	0.41688 (2)	0.588865 (9)	0.409766 (18)	0.01740 (5)	
Mo6	0.09067 (2)	0.751816 (9)	0.154478 (18)	0.01716 (5)	
Mo7	0.19104 (2)	0.507896 (9)	0.350528 (19)	0.01738 (5)	
Mo8	−0.15388 (2)	0.664402 (9)	0.090979 (19)	0.01764 (5)	
Mo9	−0.09683 (2)	0.542448 (9)	0.152746 (19)	0.01797 (5)	
Mo10	0.26798 (2)	0.673700 (9)	0.100035 (18)	0.01724 (5)	
Mo11	0.28028 (2)	0.541453 (9)	0.170021 (18)	0.01750 (5)	
Mo12	0.00156 (2)	0.596892 (9)	−0.005718 (18)	0.01759 (5)	
P1	0.13305 (6)	0.62980 (3)	0.25327 (5)	0.01579 (13)	
O1S	0.6654 (2)	0.61043 (9)	0.33985 (17)	0.0277 (5)	
O1W	0.2136 (3)	0.50927 (12)	0.6577 (2)	0.0489 (7)	
H1WA	0.2650	0.5064	0.6459	0.059*	
H1WB	0.1675	0.4947	0.6046	0.059*	
O1	0.10526 (17)	0.64338 (7)	0.34244 (15)	0.0173 (4)	
O2	0.18748 (17)	0.67467 (7)	0.22546 (15)	0.0170 (4)	
O2W	0.4173 (2)	0.51907 (9)	0.64138 (18)	0.0303 (5)	
H2WB	0.3994	0.5258	0.5811	0.036*	
H2WA	0.4423	0.4890	0.6680	0.036*	
O2S	0.0596 (2)	0.68215 (10)	0.67215 (18)	0.0321 (5)	
O3S	0.4383 (2)	0.59570 (9)	0.83909 (18)	0.0292 (5)	
O3W	0.8029 (2)	0.64000 (8)	0.71502 (17)	0.0266 (5)	
H3WA	0.8625	0.6275	0.7668	0.032*	
H3WB	0.8024	0.6671	0.7430	0.032*	
O3	0.21837 (17)	0.58625 (7)	0.28277 (15)	0.0172 (4)	
O4	0.02149 (17)	0.61520 (7)	0.16266 (15)	0.0178 (4)	
O5	0.30395 (17)	0.70902 (7)	0.43468 (15)	0.0182 (4)	
O6	0.12074 (17)	0.71361 (7)	0.46892 (15)	0.0186 (4)	
O7	0.33997 (18)	0.61763 (8)	0.49225 (15)	0.0197 (4)	
O8	0.14437 (18)	0.62037 (8)	0.52687 (15)	0.0191 (4)	
O9	−0.05958 (17)	0.65483 (8)	0.40290 (16)	0.0201 (4)	
O10	0.24744 (18)	0.76801 (8)	0.26560 (15)	0.0194 (4)	
O11	0.05341 (17)	0.74765 (8)	0.27458 (16)	0.0193 (4)	
O12	−0.11778 (17)	0.68535 (8)	0.21912 (16)	0.0198 (4)	
O13	0.42021 (17)	0.65201 (8)	0.36937 (15)	0.0188 (4)	
O14	−0.08156 (18)	0.57175 (8)	0.28521 (16)	0.0198 (4)	
O15	0.34077 (18)	0.52739 (8)	0.43200 (15)	0.0196 (4)	
O16	0.12090 (18)	0.54363 (8)	0.41412 (16)	0.0198 (4)	
O17	0.16896 (17)	0.73395 (8)	0.07979 (15)	0.0192 (4)	
O18	−0.03860 (17)	0.71521 (8)	0.09183 (15)	0.0188 (4)	
O19	0.37360 (17)	0.70610 (8)	0.21113 (15)	0.0189 (4)	
O20	0.31360 (17)	0.61041 (8)	0.14154 (15)	0.0192 (4)	
O21	−0.20133 (17)	0.60120 (8)	0.10127 (16)	0.0203 (4)	
O22	0.42056 (17)	0.55662 (8)	0.30009 (16)	0.0197 (4)	
O23	0.24697 (18)	0.49318 (8)	0.24407 (16)	0.0201 (4)	
O24	0.04070 (18)	0.51300 (8)	0.23044 (16)	0.0198 (4)	
O25	−0.11806 (17)	0.63845 (8)	−0.02211 (16)	0.0202 (4)	
O26	0.11346 (17)	0.64344 (8)	0.01772 (15)	0.0198 (4)	
O27	−0.08159 (18)	0.54429 (8)	0.03363 (16)	0.0208 (4)	
O28	0.13624 (18)	0.55233 (8)	0.07437 (15)	0.0198 (4)	
O29	0.32331 (19)	0.68249 (8)	0.63240 (16)	0.0230 (4)	
O30	−0.09283 (18)	0.75731 (8)	0.36978 (17)	0.0234 (4)	
O31	−0.05393 (19)	0.56240 (8)	0.48270 (17)	0.0231 (4)	
O32	0.47484 (18)	0.75507 (8)	0.39483 (16)	0.0215 (4)	
O33	0.55037 (18)	0.57974 (8)	0.50144 (16)	0.0221 (4)	
O34	0.04977 (18)	0.81057 (8)	0.11564 (16)	0.0218 (4)	
O35	0.1858 (2)	0.44995 (8)	0.38943 (17)	0.0239 (4)	
O36	−0.27941 (18)	0.69254 (8)	0.02173 (17)	0.0233 (4)	
O37	−0.19494 (19)	0.49750 (8)	0.13432 (17)	0.0251 (5)	
O38	0.31745 (19)	0.68375 (8)	0.01167 (16)	0.0234 (4)	
O39	0.34656 (19)	0.50846 (8)	0.11232 (17)	0.0234 (4)	
O40	−0.03200 (18)	0.57955 (8)	−0.12542 (16)	0.0221 (4)	
N1S	0.8071 (2)	0.64679 (10)	0.5249 (2)	0.0228 (5)	
H1NA	0.8169	0.6489	0.5901	0.034*	
H1NB	0.8694	0.6601	0.5191	0.034*	
H1NC	0.7995	0.6145	0.5057	0.034*	
N2S	0.1986 (3)	0.59691 (13)	0.7289 (2)	0.0327 (7)	
H2NA	0.1713	0.6109	0.6666	0.049*	
H2NB	0.1923	0.5634	0.7226	0.049*	
H2NC	0.2740	0.6053	0.7642	0.049*	
N3S	0.5835 (2)	0.59440 (11)	0.7239 (2)	0.0269 (6)	
H3NA	0.6444	0.5785	0.7203	0.040*	
H3NB	0.5606	0.6196	0.6786	0.040*	
H3NC	0.5242	0.5728	0.7097	0.040*	
C1S	0.6294 (3)	0.59620 (15)	0.2376 (3)	0.0326 (7)	
H1SA	0.6349	0.5602	0.2335	0.049*	
H1SB	0.6791	0.6120	0.2103	0.049*	
H1SC	0.5494	0.6066	0.1985	0.049*	
C2S	0.6681 (3)	0.66261 (12)	0.3497 (2)	0.0249 (6)	
H2SA	0.5915	0.6767	0.3072	0.030*	
H2SB	0.7253	0.6768	0.3278	0.030*	
C3S	0.7011 (3)	0.67455 (12)	0.4591 (2)	0.0247 (6)	
H3SA	0.7152	0.7105	0.4702	0.030*	
H3SB	0.6369	0.6656	0.4769	0.030*	
C4S	0.0660 (3)	0.73358 (14)	0.6557 (3)	0.0363 (8)	
H4SA	0.0185	0.7413	0.5848	0.054*	
H4SB	0.1463	0.7428	0.6729	0.054*	
H4SC	0.0377	0.7522	0.6982	0.054*	
C5S	0.1207 (4)	0.67011 (17)	0.7758 (3)	0.0407 (9)	
H5SA	0.0786	0.6826	0.8145	0.049*	
H5SB	0.1983	0.6853	0.8034	0.049*	
C6S	0.1303 (3)	0.61496 (16)	0.7831 (3)	0.0385 (9)	
H6SA	0.1684	0.6049	0.8547	0.046*	
H6SB	0.0523	0.6000	0.7531	0.046*	
C7S	0.4675 (3)	0.57169 (17)	0.9337 (3)	0.0417 (9)	
H7SA	0.4126	0.5449	0.9253	0.063*	
H7SB	0.4641	0.5957	0.9826	0.063*	
H7SC	0.5456	0.5581	0.9581	0.063*	
C8S	0.5176 (3)	0.63418 (12)	0.8428 (3)	0.0278 (7)	
H8SA	0.5458	0.6509	0.9090	0.033*	
H8SB	0.4766	0.6590	0.7901	0.033*	
C9S	0.6185 (3)	0.61453 (13)	0.8273 (2)	0.0266 (6)	
H9SA	0.6753	0.6414	0.8390	0.032*	
H9SB	0.6563	0.5881	0.8771	0.032*	

### Atomic displacement parameters (Å^2^)

**Table:**

	*U*^11^	*U*^22^	*U*^33^	*U*^12^	*U*^13^	*U*^23^
Mo1	0.01831 (11)	0.01785 (11)	0.01572 (11)	−0.00033 (9)	0.00683 (9)	−0.00083 (8)
Mo2	0.01774 (11)	0.01614 (11)	0.01939 (11)	0.00003 (9)	0.00838 (9)	−0.00108 (9)
Mo3	0.01888 (11)	0.01722 (11)	0.01885 (11)	−0.00036 (9)	0.00974 (9)	−0.00019 (9)
Mo4	0.01548 (10)	0.01697 (11)	0.01635 (11)	−0.00128 (8)	0.00544 (8)	−0.00015 (8)
Mo5	0.01689 (11)	0.01780 (11)	0.01664 (11)	0.00086 (9)	0.00619 (9)	0.00099 (8)
Mo6	0.01651 (11)	0.01596 (11)	0.01729 (11)	−0.00057 (8)	0.00542 (9)	0.00140 (8)
Mo7	0.01957 (11)	0.01499 (11)	0.01857 (11)	0.00056 (9)	0.00898 (9)	0.00065 (8)
Mo8	0.01530 (10)	0.01735 (11)	0.01846 (11)	−0.00049 (9)	0.00533 (9)	−0.00002 (9)
Mo9	0.01790 (11)	0.01625 (11)	0.01958 (11)	−0.00236 (9)	0.00771 (9)	−0.00150 (9)
Mo10	0.01738 (11)	0.01835 (11)	0.01584 (11)	−0.00129 (9)	0.00684 (9)	0.00073 (9)
Mo11	0.01854 (11)	0.01715 (11)	0.01760 (11)	0.00070 (9)	0.00836 (9)	−0.00062 (9)
Mo12	0.01766 (11)	0.01825 (11)	0.01574 (11)	−0.00166 (9)	0.00593 (9)	−0.00113 (9)
P1	0.0158 (3)	0.0156 (3)	0.0156 (3)	−0.0006 (2)	0.0062 (3)	−0.0002 (2)
O1S	0.0344 (12)	0.0269 (12)	0.0223 (11)	−0.0018 (10)	0.0125 (10)	−0.0006 (9)
O1W	0.0467 (17)	0.0505 (18)	0.0548 (19)	−0.0057 (14)	0.0266 (15)	−0.0035 (15)
O1	0.0180 (9)	0.0169 (9)	0.0174 (9)	−0.0008 (7)	0.0079 (8)	−0.0004 (7)
O2	0.0174 (9)	0.0163 (9)	0.0173 (9)	−0.0004 (7)	0.0071 (8)	0.0000 (7)
O2W	0.0351 (13)	0.0253 (12)	0.0232 (11)	−0.0023 (10)	0.0052 (10)	0.0055 (9)
O2S	0.0340 (13)	0.0372 (14)	0.0241 (12)	−0.0010 (11)	0.0113 (10)	−0.0041 (10)
O3S	0.0255 (11)	0.0328 (13)	0.0287 (12)	−0.0039 (10)	0.0108 (9)	−0.0011 (10)
O3W	0.0318 (12)	0.0215 (11)	0.0229 (11)	0.0059 (9)	0.0079 (9)	−0.0016 (9)
O3	0.0171 (9)	0.0179 (10)	0.0167 (9)	0.0000 (7)	0.0071 (7)	0.0005 (7)
O4	0.0180 (9)	0.0177 (10)	0.0166 (9)	−0.0012 (8)	0.0065 (8)	−0.0014 (7)
O5	0.0187 (9)	0.0171 (10)	0.0189 (10)	−0.0007 (8)	0.0081 (8)	−0.0004 (8)
O6	0.0195 (9)	0.0170 (10)	0.0194 (10)	−0.0011 (8)	0.0082 (8)	−0.0016 (8)
O7	0.0209 (10)	0.0198 (10)	0.0192 (10)	0.0004 (8)	0.0094 (8)	0.0019 (8)
O8	0.0210 (10)	0.0186 (10)	0.0189 (10)	−0.0007 (8)	0.0094 (8)	−0.0006 (8)
O9	0.0196 (10)	0.0208 (10)	0.0213 (10)	−0.0005 (8)	0.0098 (8)	−0.0005 (8)
O10	0.0198 (9)	0.0171 (10)	0.0199 (10)	−0.0007 (8)	0.0069 (8)	0.0013 (8)
O11	0.0185 (9)	0.0182 (10)	0.0212 (10)	−0.0013 (8)	0.0085 (8)	−0.0008 (8)
O12	0.0199 (10)	0.0180 (10)	0.0210 (10)	−0.0013 (8)	0.0079 (8)	−0.0008 (8)
O13	0.0185 (9)	0.0193 (10)	0.0188 (10)	−0.0014 (8)	0.0081 (8)	−0.0003 (8)
O14	0.0217 (10)	0.0181 (10)	0.0210 (10)	−0.0009 (8)	0.0104 (8)	−0.0010 (8)
O15	0.0207 (10)	0.0185 (10)	0.0194 (10)	0.0016 (8)	0.0080 (8)	0.0021 (8)
O16	0.0213 (10)	0.0202 (10)	0.0199 (10)	0.0010 (8)	0.0106 (8)	0.0006 (8)
O17	0.0168 (9)	0.0214 (10)	0.0186 (10)	−0.0004 (8)	0.0065 (8)	0.0029 (8)
O18	0.0163 (9)	0.0206 (10)	0.0176 (9)	−0.0014 (8)	0.0051 (8)	0.0005 (8)
O19	0.0188 (9)	0.0197 (10)	0.0180 (9)	−0.0020 (8)	0.0076 (8)	0.0005 (8)
O20	0.0181 (9)	0.0194 (10)	0.0208 (10)	0.0006 (8)	0.0088 (8)	0.0010 (8)
O21	0.0184 (9)	0.0199 (10)	0.0226 (10)	0.0001 (8)	0.0085 (8)	−0.0006 (8)
O22	0.0191 (9)	0.0209 (10)	0.0199 (10)	0.0008 (8)	0.0090 (8)	−0.0002 (8)
O23	0.0217 (10)	0.0177 (10)	0.0210 (10)	0.0001 (8)	0.0091 (8)	0.0006 (8)
O24	0.0217 (10)	0.0178 (10)	0.0205 (10)	−0.0007 (8)	0.0097 (8)	−0.0023 (8)
O25	0.0187 (9)	0.0200 (10)	0.0197 (10)	−0.0009 (8)	0.0057 (8)	0.0004 (8)
O26	0.0194 (10)	0.0198 (10)	0.0193 (10)	−0.0005 (8)	0.0073 (8)	0.0003 (8)
O27	0.0203 (10)	0.0196 (10)	0.0212 (10)	−0.0029 (8)	0.0073 (8)	−0.0024 (8)
O28	0.0207 (10)	0.0187 (10)	0.0190 (10)	−0.0011 (8)	0.0075 (8)	−0.0013 (8)
O29	0.0244 (10)	0.0248 (11)	0.0183 (10)	−0.0006 (9)	0.0075 (8)	−0.0020 (8)
O30	0.0213 (10)	0.0219 (11)	0.0282 (11)	0.0025 (8)	0.0115 (9)	−0.0019 (9)
O31	0.0256 (11)	0.0216 (11)	0.0269 (11)	−0.0005 (8)	0.0157 (9)	0.0024 (8)
O32	0.0183 (10)	0.0225 (11)	0.0211 (10)	−0.0040 (8)	0.0059 (8)	−0.0004 (8)
O33	0.0217 (10)	0.0218 (11)	0.0221 (10)	0.0018 (8)	0.0085 (8)	0.0024 (8)
O34	0.0219 (10)	0.0189 (10)	0.0229 (11)	−0.0001 (8)	0.0077 (8)	0.0033 (8)
O35	0.0298 (11)	0.0168 (10)	0.0279 (11)	0.0022 (8)	0.0149 (9)	0.0029 (8)
O36	0.0190 (10)	0.0212 (11)	0.0265 (11)	−0.0005 (8)	0.0062 (9)	−0.0001 (8)
O37	0.0242 (11)	0.0232 (11)	0.0277 (11)	−0.0046 (9)	0.0109 (9)	−0.0001 (9)
O38	0.0259 (11)	0.0267 (11)	0.0191 (10)	−0.0017 (9)	0.0109 (9)	0.0017 (8)
O39	0.0253 (11)	0.0231 (11)	0.0233 (11)	0.0012 (9)	0.0118 (9)	−0.0018 (8)
O40	0.0227 (10)	0.0235 (11)	0.0187 (10)	−0.0033 (8)	0.0073 (8)	−0.0034 (8)
N1S	0.0259 (13)	0.0211 (12)	0.0238 (13)	0.0005 (10)	0.0127 (10)	0.0000 (10)
N2S	0.0269 (14)	0.0486 (19)	0.0234 (14)	−0.0049 (13)	0.0114 (11)	0.0059 (13)
N3S	0.0269 (13)	0.0286 (14)	0.0244 (13)	−0.0044 (11)	0.0101 (11)	−0.0024 (11)
C1S	0.0325 (17)	0.043 (2)	0.0225 (16)	−0.0023 (15)	0.0119 (13)	−0.0041 (14)
C2S	0.0230 (14)	0.0254 (15)	0.0270 (16)	0.0038 (12)	0.0111 (12)	0.0035 (12)
C3S	0.0207 (14)	0.0240 (15)	0.0280 (16)	0.0030 (11)	0.0088 (12)	−0.0005 (12)
C4S	0.042 (2)	0.0353 (19)	0.0350 (19)	0.0091 (16)	0.0193 (16)	−0.0045 (15)
C5S	0.043 (2)	0.054 (3)	0.0225 (17)	−0.0019 (18)	0.0113 (15)	−0.0006 (16)
C6S	0.0352 (19)	0.056 (2)	0.0312 (18)	−0.0004 (17)	0.0205 (16)	0.0061 (17)
C7S	0.036 (2)	0.052 (2)	0.040 (2)	0.0060 (18)	0.0195 (17)	0.0121 (18)
C8S	0.0264 (15)	0.0236 (16)	0.0283 (16)	−0.0030 (12)	0.0065 (13)	−0.0038 (12)
C9S	0.0253 (15)	0.0278 (16)	0.0241 (15)	−0.0052 (12)	0.0078 (12)	−0.0017 (12)

### Geometric parameters (Å, °)

**Table:**

Mo1—O29	1.686 (2)	Mo11—O23	1.857 (2)
Mo1—O7	1.833 (2)	Mo11—O20	1.996 (2)
Mo1—O8	1.847 (2)	Mo11—O22	2.033 (2)
Mo1—O6	1.988 (2)	Mo11—O3	2.432 (2)
Mo1—O5	1.996 (2)	Mo12—O40	1.686 (2)
Mo1—O1	2.449 (2)	Mo12—O26	1.826 (2)
Mo2—O30	1.682 (2)	Mo12—O25	1.830 (2)
Mo2—O11	1.822 (2)	Mo12—O27	2.000 (2)
Mo2—O6	1.831 (2)	Mo12—O28	2.023 (2)
Mo2—O12	2.000 (2)	Mo12—O4	2.420 (2)
Mo2—O9	2.034 (2)	P1—O1	1.534 (2)
Mo2—O1	2.451 (2)	P1—O4	1.535 (2)
Mo3—O31	1.693 (2)	P1—O2	1.537 (2)
Mo3—O14	1.810 (2)	P1—O3	1.539 (2)
Mo3—O9	1.840 (2)	O1S—C2S	1.417 (4)
Mo3—O16	2.003 (2)	O1S—C1S	1.421 (4)
Mo3—O8	2.020 (2)	O1W—H1WA	0.7499
Mo3—O1	2.406 (2)	O1W—H1WB	0.8499
Mo4—O32	1.679 (2)	O2W—H2WB	0.8334
Mo4—O5	1.822 (2)	O2W—H2WA	0.8998
Mo4—O10	1.847 (2)	O2S—C4S	1.420 (5)
Mo4—O19	2.006 (2)	O2S—C5S	1.421 (4)
Mo4—O13	2.013 (2)	O3S—C7S	1.430 (5)
Mo4—O2	2.427 (2)	O3S—C8S	1.437 (4)
Mo5—O33	1.687 (2)	O3W—H3WA	0.8844
Mo5—O13	1.814 (2)	O3W—H3WB	0.8416
Mo5—O22	1.845 (2)	N1S—C3S	1.493 (4)
Mo5—O7	2.007 (2)	N1S—H1NA	0.9100
Mo5—O15	2.021 (2)	N1S—H1NB	0.9100
Mo5—O3	2.435 (2)	N1S—H1NC	0.9100
Mo6—O34	1.695 (2)	N2S—C6S	1.488 (5)
Mo6—O18	1.810 (2)	N2S—H2NA	0.9100
Mo6—O17	1.830 (2)	N2S—H2NB	0.9100
Mo6—O11	2.009 (2)	N2S—H2NC	0.9100
Mo6—O10	2.027 (2)	N3S—C9S	1.488 (4)
Mo6—O2	2.425 (2)	N3S—H3NA	0.9100
Mo7—O35	1.679 (2)	N3S—H3NB	0.9100
Mo7—O16	1.818 (2)	N3S—H3NC	0.9100
Mo7—O15	1.855 (2)	C1S—H1SA	0.9800
Mo7—O24	1.990 (2)	C1S—H1SB	0.9800
Mo7—O23	2.009 (2)	C1S—H1SC	0.9800
Mo7—O3	2.427 (2)	C2S—C3S	1.510 (5)
Mo8—O36	1.677 (2)	C2S—H2SA	0.9900
Mo8—O12	1.823 (2)	C2S—H2SB	0.9900
Mo8—O21	1.841 (2)	C3S—H3SA	0.9900
Mo8—O18	2.011 (2)	C3S—H3SB	0.9900
Mo8—O25	2.020 (2)	C4S—H4SA	0.9800
Mo8—O4	2.432 (2)	C4S—H4SB	0.9800
Mo9—O37	1.685 (2)	C4S—H4SC	0.9800
Mo9—O24	1.826 (2)	C5S—C6S	1.497 (6)
Mo9—O27	1.834 (2)	C5S—H5SA	0.9900
Mo9—O21	2.008 (2)	C5S—H5SB	0.9900
Mo9—O14	2.026 (2)	C6S—H6SA	0.9900
Mo9—O4	2.449 (2)	C6S—H6SB	0.9900
Mo10—O38	1.684 (2)	C7S—H7SA	0.9800
Mo10—O20	1.828 (2)	C7S—H7SB	0.9800
Mo10—O19	1.841 (2)	C7S—H7SC	0.9800
Mo10—O26	2.005 (2)	C8S—C9S	1.498 (5)
Mo10—O17	2.007 (2)	C8S—H8SA	0.9900
Mo10—O2	2.453 (2)	C8S—H8SB	0.9900
Mo11—O39	1.682 (2)	C9S—H9SA	0.9900
Mo11—O28	1.808 (2)	C9S—H9SB	0.9900
			
O29—Mo1—O7	104.01 (10)	O39—Mo11—O22	98.35 (10)
O29—Mo1—O8	101.25 (10)	O28—Mo11—O22	154.40 (9)
O7—Mo1—O8	96.84 (9)	O23—Mo11—O22	85.63 (9)
O29—Mo1—O6	99.71 (10)	O20—Mo11—O22	79.95 (8)
O7—Mo1—O6	154.74 (9)	O39—Mo11—O3	168.89 (9)
O8—Mo1—O6	86.82 (9)	O28—Mo11—O3	85.89 (8)
O29—Mo1—O5	103.06 (10)	O23—Mo11—O3	74.60 (8)
O7—Mo1—O5	85.00 (9)	O20—Mo11—O3	80.51 (8)
O8—Mo1—O5	154.40 (9)	O22—Mo11—O3	71.02 (7)
O6—Mo1—O5	81.30 (8)	O40—Mo12—O26	102.94 (10)
O29—Mo1—O1	169.06 (9)	O40—Mo12—O25	101.61 (10)
O7—Mo1—O1	86.18 (8)	O26—Mo12—O25	98.40 (9)
O8—Mo1—O1	73.17 (8)	O40—Mo12—O27	99.65 (10)
O6—Mo1—O1	70.92 (7)	O26—Mo12—O27	154.86 (9)
O5—Mo1—O1	81.51 (7)	O25—Mo12—O27	87.64 (9)
O30—Mo2—O11	104.59 (10)	O40—Mo12—O28	102.65 (10)
O30—Mo2—O6	103.53 (10)	O26—Mo12—O28	83.90 (9)
O11—Mo2—O6	98.15 (9)	O25—Mo12—O28	154.44 (9)
O30—Mo2—O12	101.41 (10)	O27—Mo12—O28	80.50 (8)
O11—Mo2—O12	85.81 (9)	O40—Mo12—O4	170.84 (9)
O6—Mo2—O12	152.80 (9)	O26—Mo12—O4	85.92 (8)
O30—Mo2—O9	98.09 (10)	O25—Mo12—O4	74.46 (8)
O11—Mo2—O9	155.42 (9)	O27—Mo12—O4	72.14 (8)
O6—Mo2—O9	85.47 (9)	O28—Mo12—O4	80.35 (8)
O12—Mo2—O9	80.45 (8)	O1—P1—O4	109.46 (11)
O30—Mo2—O1	168.59 (9)	O1—P1—O2	109.20 (11)
O11—Mo2—O1	86.77 (8)	O4—P1—O2	109.50 (11)
O6—Mo2—O1	73.20 (8)	O1—P1—O3	109.56 (11)
O12—Mo2—O1	80.22 (8)	O4—P1—O3	109.66 (11)
O9—Mo2—O1	70.93 (8)	O2—P1—O3	109.44 (11)
O31—Mo3—O14	104.55 (10)	C2S—O1S—C1S	111.1 (3)
O31—Mo3—O9	102.01 (10)	H1WA—O1W—H1WB	94.4
O14—Mo3—O9	98.86 (9)	P1—O1—Mo3	126.16 (11)
O31—Mo3—O16	100.74 (10)	P1—O1—Mo1	125.48 (11)
O14—Mo3—O16	85.53 (9)	Mo3—O1—Mo1	90.01 (7)
O9—Mo3—O16	154.89 (9)	P1—O1—Mo2	125.31 (11)
O31—Mo3—O8	98.20 (10)	Mo3—O1—Mo2	89.72 (7)
O14—Mo3—O8	155.00 (9)	Mo1—O1—Mo2	88.60 (7)
O9—Mo3—O8	86.23 (9)	P1—O2—Mo6	125.93 (11)
O16—Mo3—O8	80.12 (8)	P1—O2—Mo4	126.59 (11)
O31—Mo3—O1	169.32 (9)	Mo6—O2—Mo4	89.60 (7)
O14—Mo3—O1	86.09 (8)	P1—O2—Mo10	124.67 (11)
O9—Mo3—O1	75.01 (8)	Mo6—O2—Mo10	89.29 (7)
O16—Mo3—O1	80.68 (8)	Mo4—O2—Mo10	89.09 (6)
O8—Mo3—O1	71.51 (8)	H2WB—O2W—H2WA	123.0
O32—Mo4—O5	105.11 (10)	C4S—O2S—C5S	111.1 (3)
O32—Mo4—O10	102.00 (10)	C7S—O3S—C8S	113.6 (3)
O5—Mo4—O10	98.17 (9)	H3WA—O3W—H3WB	95.5
O32—Mo4—O19	97.57 (9)	P1—O3—Mo7	125.21 (11)
O5—Mo4—O19	154.78 (9)	P1—O3—Mo11	125.83 (11)
O10—Mo4—O19	87.57 (9)	Mo7—O3—Mo11	89.20 (7)
O32—Mo4—O13	101.14 (9)	P1—O3—Mo5	125.44 (11)
O5—Mo4—O13	84.70 (9)	Mo7—O3—Mo5	89.79 (7)
O10—Mo4—O13	155.02 (9)	Mo11—O3—Mo5	90.03 (7)
O19—Mo4—O13	80.19 (8)	P1—O4—Mo12	126.44 (11)
O32—Mo4—O2	168.64 (9)	P1—O4—Mo8	125.75 (11)
O5—Mo4—O2	86.18 (8)	Mo12—O4—Mo8	89.40 (7)
O10—Mo4—O2	74.64 (8)	P1—O4—Mo9	125.73 (11)
O19—Mo4—O2	71.62 (7)	Mo12—O4—Mo9	88.70 (6)
O13—Mo4—O2	80.85 (7)	Mo8—O4—Mo9	88.86 (7)
O33—Mo5—O13	104.03 (10)	Mo4—O5—Mo1	152.24 (12)
O33—Mo5—O22	103.62 (10)	Mo2—O6—Mo1	127.27 (11)
O13—Mo5—O22	98.57 (9)	Mo1—O7—Mo5	151.06 (12)
O33—Mo5—O7	99.53 (9)	Mo1—O8—Mo3	125.19 (11)
O13—Mo5—O7	85.16 (9)	Mo3—O9—Mo2	124.34 (11)
O22—Mo5—O7	154.82 (9)	Mo4—O10—Mo6	123.83 (11)
O33—Mo5—O15	98.38 (9)	Mo2—O11—Mo6	151.05 (12)
O13—Mo5—O15	155.00 (9)	Mo8—O12—Mo2	151.53 (12)
O22—Mo5—O15	86.63 (9)	Mo5—O13—Mo4	151.88 (12)
O7—Mo5—O15	80.29 (9)	Mo3—O14—Mo9	152.28 (12)
O33—Mo5—O3	169.49 (9)	Mo7—O15—Mo5	124.51 (11)
O13—Mo5—O3	86.47 (8)	Mo7—O16—Mo3	151.50 (12)
O22—Mo5—O3	73.85 (8)	Mo6—O17—Mo10	126.53 (11)
O7—Mo5—O3	81.59 (8)	Mo6—O18—Mo8	152.52 (12)
O15—Mo5—O3	71.42 (7)	Mo10—O19—Mo4	125.71 (11)
O34—Mo6—O18	104.83 (10)	Mo10—O20—Mo11	151.44 (12)
O34—Mo6—O17	102.49 (10)	Mo8—O21—Mo9	125.14 (11)
O18—Mo6—O17	99.49 (9)	Mo5—O22—Mo11	125.09 (11)
O34—Mo6—O11	101.12 (10)	Mo11—O23—Mo7	123.85 (11)
O18—Mo6—O11	84.88 (9)	Mo9—O24—Mo7	154.04 (12)
O17—Mo6—O11	153.95 (9)	Mo12—O25—Mo8	124.81 (11)
O34—Mo6—O10	97.34 (9)	Mo12—O26—Mo10	152.86 (12)
O18—Mo6—O10	155.24 (9)	Mo9—O27—Mo12	125.08 (11)
O17—Mo6—O10	86.11 (9)	Mo11—O28—Mo12	152.48 (12)
O11—Mo6—O10	80.11 (8)	C3S—N1S—H1NA	109.5
O34—Mo6—O2	168.59 (9)	C3S—N1S—H1NB	109.5
O18—Mo6—O2	86.49 (8)	H1NA—N1S—H1NB	109.5
O17—Mo6—O2	73.75 (8)	C3S—N1S—H1NC	109.5
O11—Mo6—O2	80.96 (8)	H1NA—N1S—H1NC	109.5
O10—Mo6—O2	71.84 (7)	H1NB—N1S—H1NC	109.5
O35—Mo7—O16	102.95 (10)	C6S—N2S—H2NA	109.5
O35—Mo7—O15	103.01 (10)	C6S—N2S—H2NB	109.5
O16—Mo7—O15	96.73 (9)	H2NA—N2S—H2NB	109.5
O35—Mo7—O24	101.70 (10)	C6S—N2S—H2NC	109.5
O16—Mo7—O24	85.09 (9)	H2NA—N2S—H2NC	109.5
O15—Mo7—O24	154.13 (9)	H2NB—N2S—H2NC	109.5
O35—Mo7—O23	98.99 (10)	C9S—N3S—H3NA	109.5
O16—Mo7—O23	156.27 (9)	C9S—N3S—H3NB	109.5
O15—Mo7—O23	86.99 (9)	H3NA—N3S—H3NB	109.5
O24—Mo7—O23	81.67 (8)	C9S—N3S—H3NC	109.5
O35—Mo7—O3	170.87 (9)	H3NA—N3S—H3NC	109.5
O16—Mo7—O3	86.08 (8)	H3NB—N3S—H3NC	109.5
O15—Mo7—O3	74.20 (8)	O1S—C1S—H1SA	109.5
O24—Mo7—O3	80.21 (8)	O1S—C1S—H1SB	109.5
O23—Mo7—O3	72.35 (8)	H1SA—C1S—H1SB	109.5
O36—Mo8—O12	104.15 (10)	O1S—C1S—H1SC	109.5
O36—Mo8—O21	101.92 (10)	H1SA—C1S—H1SC	109.5
O12—Mo8—O21	98.86 (9)	H1SB—C1S—H1SC	109.5
O36—Mo8—O18	101.87 (9)	O1S—C2S—C3S	107.7 (3)
O12—Mo8—O18	85.04 (9)	O1S—C2S—H2SA	110.2
O21—Mo8—O18	154.12 (9)	C3S—C2S—H2SA	110.2
O36—Mo8—O25	98.71 (10)	O1S—C2S—H2SB	110.2
O12—Mo8—O25	154.77 (9)	C3S—C2S—H2SB	110.2
O21—Mo8—O25	86.58 (9)	H2SA—C2S—H2SB	108.5
O18—Mo8—O25	79.89 (8)	N1S—C3S—C2S	110.5 (3)
O36—Mo8—O4	169.30 (9)	N1S—C3S—H3SA	109.6
O12—Mo8—O4	86.43 (8)	C2S—C3S—H3SA	109.6
O21—Mo8—O4	74.47 (8)	N1S—C3S—H3SB	109.6
O18—Mo8—O4	80.30 (8)	C2S—C3S—H3SB	109.6
O25—Mo8—O4	71.21 (7)	H3SA—C3S—H3SB	108.1
O37—Mo9—O24	103.97 (10)	O2S—C4S—H4SA	109.5
O37—Mo9—O27	103.70 (10)	O2S—C4S—H4SB	109.5
O24—Mo9—O27	98.31 (9)	H4SA—C4S—H4SB	109.5
O37—Mo9—O21	100.27 (10)	O2S—C4S—H4SC	109.5
O24—Mo9—O21	153.26 (9)	H4SA—C4S—H4SC	109.5
O27—Mo9—O21	86.66 (9)	H4SB—C4S—H4SC	109.5
O37—Mo9—O14	101.59 (10)	O2S—C5S—C6S	107.2 (3)
O24—Mo9—O14	83.96 (9)	O2S—C5S—H5SA	110.3
O27—Mo9—O14	153.20 (9)	C6S—C5S—H5SA	110.3
O21—Mo9—O14	80.25 (8)	O2S—C5S—H5SB	110.3
O37—Mo9—O4	171.45 (9)	C6S—C5S—H5SB	110.3
O24—Mo9—O4	84.55 (8)	H5SA—C5S—H5SB	108.5
O27—Mo9—O4	74.02 (8)	N2S—C6S—C5S	109.7 (3)
O21—Mo9—O4	71.51 (8)	N2S—C6S—H6SA	109.7
O14—Mo9—O4	79.66 (7)	C5S—C6S—H6SA	109.7
O38—Mo10—O20	103.66 (10)	N2S—C6S—H6SB	109.7
O38—Mo10—O19	103.23 (10)	C5S—C6S—H6SB	109.7
O20—Mo10—O19	98.02 (9)	H6SA—C6S—H6SB	108.2
O38—Mo10—O26	101.40 (10)	O3S—C7S—H7SA	109.5
O20—Mo10—O26	85.02 (9)	O3S—C7S—H7SB	109.5
O19—Mo10—O26	153.67 (9)	H7SA—C7S—H7SB	109.5
O38—Mo10—O17	99.94 (10)	O3S—C7S—H7SC	109.5
O20—Mo10—O17	154.49 (9)	H7SA—C7S—H7SC	109.5
O19—Mo10—O17	85.78 (9)	H7SB—C7S—H7SC	109.5
O26—Mo10—O17	80.93 (8)	O3S—C8S—C9S	112.0 (3)
O38—Mo10—O2	169.86 (9)	O3S—C8S—H8SA	109.2
O20—Mo10—O2	86.39 (8)	C9S—C8S—H8SA	109.2
O19—Mo10—O2	73.53 (8)	O3S—C8S—H8SB	109.2
O26—Mo10—O2	80.61 (8)	C9S—C8S—H8SB	109.2
O17—Mo10—O2	70.43 (7)	H8SA—C8S—H8SB	107.9
O39—Mo11—O28	105.16 (10)	N3S—C9S—C8S	112.0 (3)
O39—Mo11—O23	101.97 (10)	N3S—C9S—H9SA	109.2
O28—Mo11—O23	99.07 (9)	C8S—C9S—H9SA	109.2
O39—Mo11—O20	101.20 (10)	N3S—C9S—H9SB	109.2
O28—Mo11—O20	85.61 (9)	C8S—C9S—H9SB	109.2
O23—Mo11—O20	154.21 (9)	H9SA—C9S—H9SB	107.9
			
O4—P1—O1—Mo3	53.93 (16)	O29—Mo1—O7—Mo5	−132.4 (2)
O2—P1—O1—Mo3	173.79 (12)	O8—Mo1—O7—Mo5	124.2 (2)
O3—P1—O1—Mo3	−66.36 (15)	O6—Mo1—O7—Mo5	27.1 (4)
O4—P1—O1—Mo1	175.34 (12)	O5—Mo1—O7—Mo5	−30.2 (2)
O2—P1—O1—Mo1	−64.80 (15)	O1—Mo1—O7—Mo5	51.6 (2)
O3—P1—O1—Mo1	55.05 (16)	O33—Mo5—O7—Mo1	134.2 (2)
O4—P1—O1—Mo2	−66.75 (15)	O13—Mo5—O7—Mo1	30.8 (2)
O2—P1—O1—Mo2	53.11 (16)	O22—Mo5—O7—Mo1	−69.1 (4)
O3—P1—O1—Mo2	172.96 (12)	O15—Mo5—O7—Mo1	−128.8 (3)
O31—Mo3—O1—P1	149.9 (4)	O3—Mo5—O7—Mo1	−56.3 (2)
O14—Mo3—O1—P1	−34.78 (14)	O29—Mo1—O8—Mo3	167.10 (13)
O9—Mo3—O1—P1	−135.04 (15)	O7—Mo1—O8—Mo3	−87.09 (13)
O16—Mo3—O1—P1	51.33 (14)	O6—Mo1—O8—Mo3	67.82 (13)
O8—Mo3—O1—P1	133.95 (15)	O5—Mo1—O8—Mo3	5.6 (3)
O31—Mo3—O1—Mo1	13.9 (5)	O1—Mo1—O8—Mo3	−3.21 (11)
O14—Mo3—O1—Mo1	−170.75 (8)	O31—Mo3—O8—Mo1	−173.76 (13)
O9—Mo3—O1—Mo1	88.99 (8)	O14—Mo3—O8—Mo1	30.8 (3)
O16—Mo3—O1—Mo1	−84.65 (8)	O9—Mo3—O8—Mo1	−72.15 (14)
O8—Mo3—O1—Mo1	−2.03 (7)	O16—Mo3—O8—Mo1	86.68 (13)
O31—Mo3—O1—Mo2	−74.7 (5)	O1—Mo3—O8—Mo1	3.29 (11)
O14—Mo3—O1—Mo2	100.65 (8)	O31—Mo3—O9—Mo2	168.88 (13)
O9—Mo3—O1—Mo2	0.39 (7)	O14—Mo3—O9—Mo2	−84.07 (13)
O16—Mo3—O1—Mo2	−173.25 (8)	O16—Mo3—O9—Mo2	14.4 (3)
O8—Mo3—O1—Mo2	−90.63 (8)	O8—Mo3—O9—Mo2	71.29 (13)
O29—Mo1—O1—P1	165.2 (4)	O1—Mo3—O9—Mo2	−0.57 (11)
O7—Mo1—O1—P1	−35.90 (14)	O30—Mo2—O9—Mo3	−176.26 (13)
O8—Mo1—O1—P1	−134.24 (16)	O11—Mo2—O9—Mo3	26.5 (3)
O6—Mo1—O1—P1	133.28 (15)	O6—Mo2—O9—Mo3	−73.21 (13)
O5—Mo1—O1—P1	49.60 (14)	O12—Mo2—O9—Mo3	83.44 (13)
O29—Mo1—O1—Mo3	−58.4 (5)	O1—Mo2—O9—Mo3	0.57 (11)
O7—Mo1—O1—Mo3	100.54 (8)	O32—Mo4—O10—Mo6	166.04 (13)
O8—Mo1—O1—Mo3	2.20 (8)	O5—Mo4—O10—Mo6	−86.52 (13)
O6—Mo1—O1—Mo3	−90.28 (8)	O19—Mo4—O10—Mo6	68.81 (13)
O5—Mo1—O1—Mo3	−173.96 (8)	O13—Mo4—O10—Mo6	8.5 (3)
O29—Mo1—O1—Mo2	31.4 (5)	O2—Mo4—O10—Mo6	−2.80 (11)
O7—Mo1—O1—Mo2	−169.74 (8)	O34—Mo6—O10—Mo4	−173.43 (13)
O8—Mo1—O1—Mo2	91.92 (8)	O18—Mo6—O10—Mo4	33.0 (3)
O6—Mo1—O1—Mo2	−0.56 (7)	O17—Mo6—O10—Mo4	−71.32 (13)
O5—Mo1—O1—Mo2	−84.24 (7)	O11—Mo6—O10—Mo4	86.49 (13)
O30—Mo2—O1—P1	151.7 (4)	O2—Mo6—O10—Mo4	2.84 (11)
O11—Mo2—O1—P1	−33.84 (14)	O30—Mo2—O11—Mo6	−130.3 (2)
O6—Mo2—O1—P1	−133.36 (15)	O6—Mo2—O11—Mo6	123.3 (2)
O12—Mo2—O1—P1	52.46 (14)	O12—Mo2—O11—Mo6	−29.6 (2)
O9—Mo2—O1—P1	135.66 (15)	O9—Mo2—O11—Mo6	26.4 (4)
O30—Mo2—O1—Mo3	15.7 (5)	O1—Mo2—O11—Mo6	50.8 (2)
O11—Mo2—O1—Mo3	−169.86 (8)	O34—Mo6—O11—Mo2	134.8 (2)
O6—Mo2—O1—Mo3	90.62 (8)	O18—Mo6—O11—Mo2	30.7 (2)
O12—Mo2—O1—Mo3	−83.56 (8)	O17—Mo6—O11—Mo2	−70.5 (3)
O9—Mo2—O1—Mo3	−0.36 (7)	O10—Mo6—O11—Mo2	−129.5 (3)
O30—Mo2—O1—Mo1	−74.3 (5)	O2—Mo6—O11—Mo2	−56.6 (2)
O11—Mo2—O1—Mo1	100.12 (8)	O36—Mo8—O12—Mo2	−128.3 (2)
O6—Mo2—O1—Mo1	0.60 (7)	O21—Mo8—O12—Mo2	126.9 (2)
O12—Mo2—O1—Mo1	−173.57 (8)	O18—Mo8—O12—Mo2	−27.3 (2)
O9—Mo2—O1—Mo1	−90.38 (8)	O25—Mo8—O12—Mo2	26.0 (4)
O1—P1—O2—Mo6	−67.43 (15)	O4—Mo8—O12—Mo2	53.3 (2)
O4—P1—O2—Mo6	52.41 (16)	O30—Mo2—O12—Mo8	132.5 (3)
O3—P1—O2—Mo6	172.64 (12)	O11—Mo2—O12—Mo8	28.4 (3)
O1—P1—O2—Mo4	54.39 (16)	O6—Mo2—O12—Mo8	−71.3 (3)
O4—P1—O2—Mo4	174.23 (12)	O9—Mo2—O12—Mo8	−131.1 (3)
O3—P1—O2—Mo4	−65.54 (16)	O1—Mo2—O12—Mo8	−59.0 (2)
O1—P1—O2—Mo10	173.70 (11)	O33—Mo5—O13—Mo4	−128.7 (2)
O4—P1—O2—Mo10	−66.46 (15)	O22—Mo5—O13—Mo4	124.8 (2)
O3—P1—O2—Mo10	53.77 (15)	O7—Mo5—O13—Mo4	−30.1 (2)
O34—Mo6—O2—P1	154.2 (4)	O15—Mo5—O13—Mo4	24.3 (4)
O18—Mo6—O2—P1	−32.65 (14)	O3—Mo5—O13—Mo4	51.7 (2)
O17—Mo6—O2—P1	−133.60 (15)	O32—Mo4—O13—Mo5	135.1 (2)
O11—Mo6—O2—P1	52.71 (14)	O5—Mo4—O13—Mo5	30.7 (2)
O10—Mo6—O2—P1	135.19 (15)	O10—Mo4—O13—Mo5	−67.2 (4)
O34—Mo6—O2—Mo4	17.2 (5)	O19—Mo4—O13—Mo5	−129.0 (3)
O18—Mo6—O2—Mo4	−169.64 (8)	O2—Mo4—O13—Mo5	−56.2 (2)
O17—Mo6—O2—Mo4	89.42 (8)	O31—Mo3—O14—Mo9	−127.3 (3)
O11—Mo6—O2—Mo4	−84.27 (7)	O9—Mo3—O14—Mo9	127.7 (3)
O10—Mo6—O2—Mo4	−1.80 (7)	O16—Mo3—O14—Mo9	−27.4 (3)
O34—Mo6—O2—Mo10	−71.9 (5)	O8—Mo3—O14—Mo9	27.6 (4)
O18—Mo6—O2—Mo10	101.27 (8)	O1—Mo3—O14—Mo9	53.6 (3)
O17—Mo6—O2—Mo10	0.32 (7)	O37—Mo9—O14—Mo3	130.0 (3)
O11—Mo6—O2—Mo10	−173.37 (8)	O24—Mo9—O14—Mo3	27.0 (3)
O10—Mo6—O2—Mo10	−90.89 (8)	O27—Mo9—O14—Mo3	−69.5 (3)
O32—Mo4—O2—P1	151.3 (4)	O21—Mo9—O14—Mo3	−131.4 (3)
O5—Mo4—O2—P1	−35.03 (14)	O4—Mo9—O14—Mo3	−58.6 (3)
O10—Mo4—O2—P1	−134.58 (16)	O35—Mo7—O15—Mo5	168.30 (13)
O19—Mo4—O2—P1	132.84 (15)	O16—Mo7—O15—Mo5	−86.70 (14)
O13—Mo4—O2—P1	50.22 (14)	O24—Mo7—O15—Mo5	5.9 (3)
O32—Mo4—O2—Mo6	−72.2 (5)	O23—Mo7—O15—Mo5	69.77 (13)
O5—Mo4—O2—Mo6	101.50 (8)	O3—Mo7—O15—Mo5	−2.73 (11)
O10—Mo4—O2—Mo6	1.94 (8)	O33—Mo5—O15—Mo7	−174.61 (13)
O19—Mo4—O2—Mo6	−90.63 (8)	O13—Mo5—O15—Mo7	31.8 (3)
O13—Mo4—O2—Mo6	−173.26 (8)	O22—Mo5—O15—Mo7	−71.32 (14)
O32—Mo4—O2—Mo10	17.1 (5)	O7—Mo5—O15—Mo7	87.07 (13)
O5—Mo4—O2—Mo10	−169.20 (8)	O3—Mo5—O15—Mo7	2.76 (11)
O10—Mo4—O2—Mo10	91.24 (8)	O35—Mo7—O16—Mo3	−128.1 (3)
O19—Mo4—O2—Mo10	−1.33 (7)	O15—Mo7—O16—Mo3	126.8 (3)
O13—Mo4—O2—Mo10	−83.96 (7)	O24—Mo7—O16—Mo3	−27.2 (2)
O38—Mo10—O2—P1	153.2 (5)	O23—Mo7—O16—Mo3	29.0 (4)
O20—Mo10—O2—P1	−34.65 (14)	O3—Mo7—O16—Mo3	53.3 (2)
O19—Mo10—O2—P1	−134.12 (15)	O31—Mo3—O16—Mo7	132.5 (3)
O26—Mo10—O2—P1	50.90 (13)	O14—Mo3—O16—Mo7	28.5 (3)
O17—Mo10—O2—P1	134.53 (15)	O9—Mo3—O16—Mo7	−72.8 (4)
O38—Mo10—O2—Mo6	18.4 (5)	O8—Mo3—O16—Mo7	−130.9 (3)
O20—Mo10—O2—Mo6	−169.48 (8)	O1—Mo3—O16—Mo7	−58.2 (2)
O19—Mo10—O2—Mo6	91.05 (8)	O34—Mo6—O17—Mo10	168.39 (13)
O26—Mo10—O2—Mo6	−83.94 (8)	O18—Mo6—O17—Mo10	−83.98 (14)
O17—Mo10—O2—Mo6	−0.30 (7)	O11—Mo6—O17—Mo10	13.8 (3)
O38—Mo10—O2—Mo4	−71.2 (5)	O10—Mo6—O17—Mo10	71.72 (13)
O20—Mo10—O2—Mo4	100.91 (8)	O2—Mo6—O17—Mo10	−0.49 (11)
O19—Mo10—O2—Mo4	1.44 (7)	O38—Mo10—O17—Mo6	−176.22 (13)
O26—Mo10—O2—Mo4	−173.55 (8)	O20—Mo10—O17—Mo6	26.3 (3)
O17—Mo10—O2—Mo4	−89.91 (8)	O19—Mo10—O17—Mo6	−73.51 (14)
O1—P1—O3—Mo7	53.66 (16)	O26—Mo10—O17—Mo6	83.69 (14)
O4—P1—O3—Mo7	−66.51 (15)	O2—Mo10—O17—Mo6	0.49 (11)
O2—P1—O3—Mo7	173.36 (11)	O34—Mo6—O18—Mo8	−130.5 (3)
O1—P1—O3—Mo11	172.90 (12)	O17—Mo6—O18—Mo8	123.8 (3)
O4—P1—O3—Mo11	52.74 (16)	O11—Mo6—O18—Mo8	−30.3 (3)
O2—P1—O3—Mo11	−67.39 (15)	O10—Mo6—O18—Mo8	22.3 (4)
O1—P1—O3—Mo5	−66.13 (15)	O2—Mo6—O18—Mo8	50.9 (3)
O4—P1—O3—Mo5	173.71 (12)	O36—Mo8—O18—Mo6	133.8 (3)
O2—P1—O3—Mo5	53.58 (16)	O12—Mo8—O18—Mo6	30.4 (3)
O16—Mo7—O3—P1	−34.99 (14)	O21—Mo8—O18—Mo6	−69.7 (4)
O15—Mo7—O3—P1	−133.13 (15)	O25—Mo8—O18—Mo6	−129.3 (3)
O24—Mo7—O3—P1	50.70 (14)	O4—Mo8—O18—Mo6	−56.8 (3)
O23—Mo7—O3—P1	134.99 (15)	O38—Mo10—O19—Mo4	167.91 (13)
O16—Mo7—O3—Mo11	−169.97 (8)	O20—Mo10—O19—Mo4	−85.94 (14)
O15—Mo7—O3—Mo11	91.90 (8)	O26—Mo10—O19—Mo4	9.1 (3)
O24—Mo7—O3—Mo11	−84.28 (8)	O17—Mo10—O19—Mo4	68.68 (13)
O23—Mo7—O3—Mo11	0.02 (7)	O2—Mo10—O19—Mo4	−2.14 (11)
O16—Mo7—O3—Mo5	100.00 (8)	O32—Mo4—O19—Mo10	−174.21 (14)
O15—Mo7—O3—Mo5	1.87 (8)	O5—Mo4—O19—Mo10	31.7 (3)
O24—Mo7—O3—Mo5	−174.31 (8)	O10—Mo4—O19—Mo10	−72.42 (14)
O23—Mo7—O3—Mo5	−90.01 (8)	O13—Mo4—O19—Mo10	85.72 (13)
O39—Mo11—O3—P1	152.1 (4)	O2—Mo4—O19—Mo10	2.19 (11)
O28—Mo11—O3—P1	−33.96 (14)	O38—Mo10—O20—Mo11	−128.0 (2)
O23—Mo11—O3—P1	−134.54 (15)	O19—Mo10—O20—Mo11	126.2 (2)
O20—Mo11—O3—P1	52.26 (14)	O26—Mo10—O20—Mo11	−27.5 (2)
O22—Mo11—O3—P1	134.82 (15)	O17—Mo10—O20—Mo11	29.2 (4)
O39—Mo11—O3—Mo7	−73.4 (5)	O2—Mo10—O20—Mo11	53.4 (2)
O28—Mo11—O3—Mo7	100.56 (8)	O39—Mo11—O20—Mo10	132.5 (2)
O23—Mo11—O3—Mo7	−0.02 (7)	O28—Mo11—O20—Mo10	27.9 (2)
O20—Mo11—O3—Mo7	−173.21 (8)	O23—Mo11—O20—Mo10	−73.8 (3)
O22—Mo11—O3—Mo7	−90.66 (8)	O22—Mo11—O20—Mo10	−130.8 (3)
O39—Mo11—O3—Mo5	16.4 (5)	O3—Mo11—O20—Mo10	−58.6 (2)
O28—Mo11—O3—Mo5	−169.65 (8)	O36—Mo8—O21—Mo9	168.30 (13)
O23—Mo11—O3—Mo5	89.77 (8)	O12—Mo8—O21—Mo9	−85.09 (14)
O20—Mo11—O3—Mo5	−83.42 (8)	O18—Mo8—O21—Mo9	11.8 (3)
O22—Mo11—O3—Mo5	−0.87 (7)	O25—Mo8—O21—Mo9	70.12 (13)
O33—Mo5—O3—P1	147.5 (4)	O4—Mo8—O21—Mo9	−1.33 (11)
O13—Mo5—O3—P1	−35.04 (14)	O37—Mo9—O21—Mo8	−176.25 (14)
O22—Mo5—O3—P1	−135.02 (15)	O24—Mo9—O21—Mo8	28.9 (3)
O7—Mo5—O3—P1	50.58 (14)	O27—Mo9—O21—Mo8	−72.92 (14)
O15—Mo5—O3—P1	133.10 (15)	O14—Mo9—O21—Mo8	83.60 (14)
O33—Mo5—O3—Mo7	12.7 (5)	O4—Mo9—O21—Mo8	1.34 (11)
O13—Mo5—O3—Mo7	−169.88 (8)	O33—Mo5—O22—Mo11	168.07 (13)
O22—Mo5—O3—Mo7	90.14 (8)	O13—Mo5—O22—Mo11	−85.14 (14)
O7—Mo5—O3—Mo7	−84.26 (8)	O7—Mo5—O22—Mo11	11.7 (3)
O15—Mo5—O3—Mo7	−1.74 (7)	O15—Mo5—O22—Mo11	70.25 (13)
O33—Mo5—O3—Mo11	−76.5 (5)	O3—Mo5—O22—Mo11	−1.37 (11)
O13—Mo5—O3—Mo11	100.92 (8)	O39—Mo11—O22—Mo5	−175.29 (14)
O22—Mo5—O3—Mo11	0.94 (8)	O28—Mo11—O22—Mo5	28.1 (3)
O7—Mo5—O3—Mo11	−173.46 (8)	O23—Mo11—O22—Mo5	−73.81 (14)
O15—Mo5—O3—Mo11	−90.94 (8)	O20—Mo11—O22—Mo5	84.73 (13)
O1—P1—O4—Mo12	174.12 (12)	O3—Mo11—O22—Mo5	1.40 (11)
O2—P1—O4—Mo12	54.44 (16)	O39—Mo11—O23—Mo7	169.15 (13)
O3—P1—O4—Mo12	−65.66 (16)	O28—Mo11—O23—Mo7	−83.13 (13)
O1—P1—O4—Mo8	53.07 (16)	O20—Mo11—O23—Mo7	15.6 (3)
O2—P1—O4—Mo8	−66.61 (16)	O22—Mo11—O23—Mo7	71.52 (13)
O3—P1—O4—Mo8	173.30 (12)	O3—Mo11—O23—Mo7	0.03 (11)
O1—P1—O4—Mo9	−66.12 (16)	O35—Mo7—O23—Mo11	−177.09 (13)
O2—P1—O4—Mo9	174.20 (12)	O16—Mo7—O23—Mo11	25.5 (3)
O3—P1—O4—Mo9	54.11 (16)	O15—Mo7—O23—Mo11	−74.39 (13)
O26—Mo12—O4—P1	−33.80 (15)	O24—Mo7—O23—Mo11	82.29 (13)
O25—Mo12—O4—P1	−133.70 (16)	O3—Mo7—O23—Mo11	−0.03 (11)
O27—Mo12—O4—P1	133.73 (16)	O37—Mo9—O24—Mo7	−126.3 (3)
O28—Mo12—O4—P1	50.69 (14)	O27—Mo9—O24—Mo7	127.3 (3)
O26—Mo12—O4—Mo8	102.14 (8)	O21—Mo9—O24—Mo7	28.1 (4)
O25—Mo12—O4—Mo8	2.24 (8)	O14—Mo9—O24—Mo7	−25.8 (3)
O27—Mo12—O4—Mo8	−90.32 (8)	O4—Mo9—O24—Mo7	54.3 (3)
O28—Mo12—O4—Mo8	−173.36 (8)	O35—Mo7—O24—Mo9	129.2 (3)
O26—Mo12—O4—Mo9	−168.99 (8)	O16—Mo7—O24—Mo9	26.9 (3)
O25—Mo12—O4—Mo9	91.12 (8)	O15—Mo7—O24—Mo9	−68.4 (4)
O27—Mo12—O4—Mo9	−1.45 (7)	O23—Mo7—O24—Mo9	−133.3 (3)
O28—Mo12—O4—Mo9	−84.49 (8)	O3—Mo7—O24—Mo9	−59.9 (3)
O36—Mo8—O4—P1	154.5 (4)	O40—Mo12—O25—Mo8	168.20 (13)
O12—Mo8—O4—P1	−33.75 (14)	O26—Mo12—O25—Mo8	−86.65 (14)
O21—Mo8—O4—P1	−133.97 (16)	O27—Mo12—O25—Mo8	68.83 (13)
O18—Mo8—O4—P1	51.82 (14)	O28—Mo12—O25—Mo8	6.8 (3)
O25—Mo8—O4—P1	134.36 (16)	O4—Mo12—O25—Mo8	−3.29 (11)
O36—Mo8—O4—Mo12	18.1 (5)	O36—Mo8—O25—Mo12	−172.97 (13)
O12—Mo8—O4—Mo12	−170.17 (8)	O12—Mo8—O25—Mo12	32.2 (3)
O21—Mo8—O4—Mo12	89.60 (8)	O21—Mo8—O25—Mo12	−71.42 (14)
O18—Mo8—O4—Mo12	−84.61 (8)	O18—Mo8—O25—Mo12	86.42 (13)
O25—Mo8—O4—Mo12	−2.07 (7)	O4—Mo8—O25—Mo12	3.33 (11)
O36—Mo8—O4—Mo9	−70.7 (5)	O40—Mo12—O26—Mo10	−131.5 (3)
O12—Mo8—O4—Mo9	101.11 (8)	O25—Mo12—O26—Mo10	124.5 (3)
O21—Mo8—O4—Mo9	0.89 (8)	O27—Mo12—O26—Mo10	21.9 (4)
O18—Mo8—O4—Mo9	−173.32 (8)	O28—Mo12—O26—Mo10	−29.9 (3)
O25—Mo8—O4—Mo9	−90.78 (8)	O4—Mo12—O26—Mo10	50.8 (3)
O24—Mo9—O4—P1	−33.86 (14)	O38—Mo10—O26—Mo12	133.6 (3)
O27—Mo9—O4—P1	−134.13 (16)	O20—Mo10—O26—Mo12	30.7 (3)
O21—Mo9—O4—P1	134.05 (15)	O19—Mo10—O26—Mo12	−67.4 (4)
O14—Mo9—O4—P1	50.97 (14)	O17—Mo10—O26—Mo12	−128.0 (3)
O24—Mo9—O4—Mo12	101.83 (8)	O2—Mo10—O26—Mo12	−56.5 (3)
O27—Mo9—O4—Mo12	1.56 (8)	O37—Mo9—O27—Mo12	169.17 (13)
O21—Mo9—O4—Mo12	−90.26 (8)	O24—Mo9—O27—Mo12	−84.17 (14)
O14—Mo9—O4—Mo12	−173.33 (8)	O21—Mo9—O27—Mo12	69.40 (13)
O24—Mo9—O4—Mo8	−168.74 (8)	O14—Mo9—O27—Mo12	8.9 (3)
O27—Mo9—O4—Mo8	90.99 (8)	O4—Mo9—O27—Mo12	−2.31 (11)
O21—Mo9—O4—Mo8	−0.83 (7)	O40—Mo12—O27—Mo9	−173.45 (14)
O14—Mo9—O4—Mo8	−83.91 (8)	O26—Mo12—O27—Mo9	32.8 (3)
O32—Mo4—O5—Mo1	−130.9 (2)	O25—Mo12—O27—Mo9	−72.06 (14)
O10—Mo4—O5—Mo1	124.2 (2)	O28—Mo12—O27—Mo9	85.21 (14)
O19—Mo4—O5—Mo1	22.4 (4)	O4—Mo12—O27—Mo9	2.36 (11)
O13—Mo4—O5—Mo1	−30.8 (2)	O39—Mo11—O28—Mo12	−128.7 (3)
O2—Mo4—O5—Mo1	50.4 (2)	O23—Mo11—O28—Mo12	126.2 (3)
O29—Mo1—O5—Mo4	135.0 (2)	O20—Mo11—O28—Mo12	−28.3 (3)
O7—Mo1—O5—Mo4	31.8 (2)	O22—Mo11—O28—Mo12	27.3 (4)
O8—Mo1—O5—Mo4	−63.6 (4)	O3—Mo11—O28—Mo12	52.5 (3)
O6—Mo1—O5—Mo4	−126.9 (3)	O40—Mo12—O28—Mo11	131.1 (3)
O1—Mo1—O5—Mo4	−55.1 (2)	O26—Mo12—O28—Mo11	29.2 (3)
O30—Mo2—O6—Mo1	167.74 (13)	O25—Mo12—O28—Mo11	−67.5 (4)
O11—Mo2—O6—Mo1	−85.03 (14)	O27—Mo12—O28—Mo11	−131.0 (3)
O12—Mo2—O6—Mo1	11.7 (3)	O4—Mo12—O28—Mo11	−57.7 (3)
O9—Mo2—O6—Mo1	70.50 (14)	C1S—O1S—C2S—C3S	177.2 (3)
O1—Mo2—O6—Mo1	−0.93 (12)	O1S—C2S—C3S—N1S	50.8 (3)
O29—Mo1—O6—Mo2	−173.21 (14)	C4S—O2S—C5S—C6S	−168.4 (3)
O7—Mo1—O6—Mo2	27.0 (3)	O2S—C5S—C6S—N2S	62.7 (4)
O8—Mo1—O6—Mo2	−72.34 (14)	C7S—O3S—C8S—C9S	86.4 (4)
O5—Mo1—O6—Mo2	84.91 (14)	O3S—C8S—C9S—N3S	66.0 (4)
O1—Mo1—O6—Mo2	0.94 (12)		

### Hydrogen-bond geometry (Å, °)

**Table:**

*D*—H···*A*	*D*—H	H···*A*	*D*···*A*	*D*—H···*A*
O1W—H1WA···O2W	0.75	2.00	2.726 (5)	161
O1W—H1WB···O31^i^	0.85	2.14	2.936 (4)	157
O2W—H2WB···O15	0.83	1.99	2.807 (3)	167
O2W—H2WA···O22^ii^	0.90	2.03	2.780 (3)	140
O3W—H3WA···O40^iii^	0.88	2.05	2.904 (3)	162
O3W—H3WB···O10^iv^	0.84	1.97	2.772 (3)	159
N1S—H1NA···O3W	0.91	1.93	2.812 (4)	164
N1S—H1NB···O2S^v^	0.91	2.59	3.182 (4)	123
N1S—H1NB···O9^v^	0.91	2.24	2.949 (4)	134
N1S—H1NC···O1S	0.91	2.31	2.731 (4)	108
N1S—H1NC···O31^v^	0.91	2.48	3.110 (4)	127
N1S—H1NC···O35^ii^	0.91	2.28	2.887 (3)	124
N2S—H2NA···O2S	0.91	2.42	2.814 (5)	106
N2S—H2NA···O8	0.91	1.94	2.811 (3)	159
N2S—H2NB···O1W	0.91	1.83	2.629 (5)	145
N2S—H2NC···O3S	0.91	1.93	2.798 (5)	158
N3S—H3NA···O23^ii^	0.91	2.31	3.110 (4)	146
N3S—H3NB···O34^iv^	0.91	2.08	2.955 (4)	161
N3S—H3NC···O2W	0.91	1.96	2.822 (4)	158

Symmetry codes:  (i) −*x*, −*y*+1, −*z*+1; (ii) −*x*+1, −*y*+1, −*z*+1; (iii) *x*+1, *y*, *z*+1; (iv) *x*+1/2, −*y*+3/2, *z*+1/2; (v) *x*+1, *y*, *z*.
